# Supplementary material for: A model of flux regulation in the cholesterol biosynthesis pathway: Immune mediated graduated flux reduction versus statin-like led stepped flux reduction
Source: Biochimie. 2013 Mar;95(3):613–21. doi: 10.1016/j.biochi.2012.05.024 (PMC3585962; doi:10.1016/j.biochi.2012.05.024)
Supplement: Supplementary file 1 [file mmc1.pdf]

Supplementary Material - A model of flux regulation in the cholesterol biosynthesis pathway: Immune mediated graduated flux reduction versus statin-like led stepped flux reduction

Steven Watterson[ab#], Maria Luisa Guerriero[bc1], Mathieu Blanc[ad],
Alexander Mazein[ab], Laurence Loewe[bc2], Kevin A Robertson[ab],
Holly Gibbs[a3], Guanghou Shui[e], Markus R Wenk[e],
Jane Hillston[bc] and Peter Ghazal[ab#]

February 6, 2012

[a] Division of Pathway Medicine, University of Edinburgh Medical, Chancellors Building, 49 Little France Crescent, Edinburgh EH16 4SB, Scotland, United Kingdom

[b] Centre for Systems Biology at Edinburgh, CH Waddington Building, The King's Buildings, West Mains Road, Edinburgh, EH9 3JU, Scotland, United Kingdom

[c] School of Informatics, Informatics Forum, 10 Crichton Street, University of Edinburgh, EH8 9AB, Scotland, United Kingdom

[d] Centre for Cardiovascular Science, University of Edinburgh, QMRI, 49 Little France Crescent, EH16 4TJ, Scotland, United Kingdom

[e] Department of Biochemistry and Department of Biological Sciences, National University of Singapore, Singapore 117597

PRESENT ADDRESSES:-

[1] Systems Biology Ireland, Conway Institute, University College Dublin Belfield, Dublin 4, Ireland

[2] Wisconsin Institute for Discovery, 330 North Orchard Street, University of Wisconsin-Madison, Madison, WI 53715, USA

[3] Tissue Microscopy Laboratory, Department of Biomedical Engineering, 337 Zachry Engineering Center, 3120 Texas A&M University, College Station, TX 77843, USA

[#] Corresponding authors:-
s.watterson@ed.ac.uk, ph:+44 131 2426242, f: +44 131 2426244
p.ghazal@ed.ac.uk, ph: +44 131 2426242, f: +44 131 2426244

1 Abbreviations used in Fig. 1A.

Acronym	Metabolite
ACoA	Acetyl-CoA
HCoA	HMG-CoA
M	Mevalonate
M5P	Mevalonate-5P
M5PP	Mevalonate-5PP
IsPP	Isopentyl-PP
FPP	Farnesyl-PP
Squa	Squalene
23Ox	2,3 oxydosqualene
Lan	Lanosterol
44Di	4,4 dimethyl-cholesta-8,14,24-trienol
14De	14-demethyl-lanosterol
4MZC	4-methylzymosterol-carboxylate
3K4M	3-keto-4-methyl-zymosterol
4MZ	4-methylzymosterol
Zym	Zymosterol
Cho7	Cholesta-7,24-dien-3beta-ol
7DeD	7-dehydro-desmosterol
Des	Desmosterol
Cho8	Cholesta-8,en-3beta-ol
Lath	Lathosterol
7DeC	7 dehydro-cholesterol
Chol	Cholesterol

2 Relating Michaelis-Menten interactions to Mass-action interactions

For a Michaelis-Menten interaction of the form

$$\frac{dP}{dt} = \frac{k_{cat}ES}{k_m + S}$$

where k_{cat} is the turnover parameter, k_m is the Michaelis-Menten constant, E is the enzyme concentration and S is the substrate concentration, we want to investigate the consequences of S being small (ie much less that k_m). If we define a small value for S as ΔS (where $\Delta S << k_m$), we have

$$\frac{dP}{dt} = \frac{k_{cat}E\Delta S}{k_m + \Delta S}$$

Dividing the numerator and denominator by k_m gives

$$\frac{dP}{dt} = \frac{\frac{k_{cat}}{k_m}E\Delta S}{1 + \frac{\Delta S}{k_m}}$$

We have $\frac{\Delta S}{k_m} << 1$ and so we can use the identity that

$$\frac{1}{1-x} = 1 + x + x^2 + x^3 + x^4 + ... \qquad \text{for } |x| < 1$$

Thus

$$\frac{dP}{dt} = \frac{\frac{k_{cat}}{k_m}E\Delta S}{1 + \frac{\Delta S}{k_m}} = \frac{k_{cat}}{k_m}E\Delta S\left(1 - \frac{\Delta S}{k_m} + \left(\frac{\Delta S}{k_m}\right)^2 - \left(\frac{\Delta S}{k_m}\right)^3 + \left(\frac{\Delta S}{k_m}\right)^4 - ...\right)$$

To second order in ΔS, this is

$$\frac{dP}{dt} = \frac{k_{cat}}{k_m}E\Delta S - \frac{k_{cat}}{(k_m)^2}E(\Delta S)^2 + ...$$

As $\frac{\Delta S}{k_m} \to 0$, all but the first order term make negligible contributions, meaning that

$$\lim_{S\to 0}\frac{dP}{dt} \approx \frac{k_{cat}}{k_m}ES$$

3 Parameters gathered from the Brenda enzyme database and used in our simulations

Interaction	Turnover (hr^{-1}) / (pubmed ID)	Michaelis-Menten Constant (mM) / (pubmed ID)
ACoA-HCoA	33.48/(15233626)	—
HCoA-M	—	0.07/(16128575)
M-M5P	—	0.024/(9325256)
M5P-M5PP	36720/(6248101)	0.025/(9325256)
M5PP-IsPP	17640/(15709780)	0.0074/(9325256)
FPP-Squa	1908/(8239656)	0.0023/(9473303)
Squa-23Ox	65.88/(10666321)	7.7/(10666321)
23Ox-Lan	—	0.015/(1429550)
Lan-44Di	—	0.005/(1567403)
44Di-14De	—	0.0333/(6208195)
4MZC-3K4M	—	0.007/(4401584)
3K4M-4MZ	177.48/(14672942)	0.236/(6946726)
Zym-Cho8	—	0.037/(9291139)
Zym-Cho7	1522.8/(12133002)	0.05/(12133002)
Cho8-Lath	5122.8/(12133003)	—
Lath-7DeC	—	0.032/(3997841)
7DeC-Chol	—	1.1/(14453564)
Mean	7899	0.042 (excluding 7.7 and 1.14 as outliers)

Retrieved 27th October, 2009.

4 Estimated parameters

Interaction	Turnover (hr^{-1}) / (pubmed ID)	Michaelis-Menten Constant (mM) / (pubmed ID)
ACoA-HCoA	—	0.042
HCoA-M	7900	—
M-M5P	7900	—
IsPP-FPP	7900	0.042
23Ox-Lan	7900	—
Lan-44Di	7900	—
44Di-14De	7900	—
14De-4MZC	7900	0.042
4MZC-3K4M	7900	—
Des-Chol	7900	0.042
Zym-Cho8	7900	—
Cho8-Lath	—	0.042
Lath-7DeC	7900	—
7DeC-Chol	7900	—

Interaction	Mass action constant (hr^{-1})
4MZ-Zym	156
Cho7-7DeD	156
7DeD-Des	156

Interaction	Rate constant of competing interaction (hr^{-1}) - IFNγ treatment
ACoA-HCoA	0.0134
HCoA-M	0.1977
M-M5P	0.1858
M5P-M5PP	8.8154
M5PP-IsPP	2.0107
IsPP-FPP	8.6358
FPP-Squa	15.5664
Squa-23Ox	0.0004
23Ox-Lan	0.3178
Lan-44Di	1.1174
44Di-14De	0.6450
14De-4MZC	6.7600
4MZC-3K4M	0.5712
3K4M-4MZ	0.0011
4MZ-Zym	1.5600
Zym-Cho7	0.2680
Cho7-7DeD	1.5600
7DeD-Des	1.5600
Des-Chol	0.1855
Zym-Cho8	0.2680
Cho8-Lath	1.3027
Lath-7DeC	1.3936
7DeC-Chol	0.0296

Interaction	Rate constant of competing interaction (hr^{-1}) - mCMV infection
ACoA-HCoA	0.00745
HCoA-M	0.18893
M-M5P	0.16232
M5P-M5PP	8.32940
M5PP-IsPP	1.71684
IsPP-FPP	8.57849
FPP-Squa	18.43532
Squa-23Ox	0.00003
23Ox-Lan	0.26313
Lan-44Di	0.77913
44Di-14De	0.60956
14De-4MZC	5.82151
4MZC-3K4M	0.31487
3K4M-4MZ	0.00127
4MZ-Zym	1.56000
Zym-Cho7	0.2008
Cho7-7DeD	1.56000
7DeD-Des	1.56000
Des-Chol	0.16598
Zym-Cho8	0.2008
Cho8-Lath	0.85392
Lath-7DeC	0.96270
7DeC-Chol	0.02307

5 Normalized enzyme activity time course following infection with mCMV.

Time (hr)	HMGCR	MVK	PMVK	MVD	FDPS	FDFT1	SQLE	DHCR7
0	258	76	874	111	7029	3425	486	495
0.5	253	68	855	106	6990	3322	428	436
1	255	54	790	91	6568	3509	490	339
1.5	271	38	735	76	6036	3346	515	279
2	267	35	639	65	5082	2942	530	297
2.5	271	39	590	70	4612	2411	483	280
3	259	47	541	66	4186	1861	462	259
3.5	287	44	524	64	4387	1674	466	175
4	293	50	515	68	4666	1445	396	215
4.5	314	51	501	74	5062	1341	375	231
5	304	66	522	80	4848	1154	304	296
5.5	304	64	535	71	4404	1022	309	322
6	290	77	550	73	3468	997	282	334
6.5	307	63	574	65	3335	1029	318	314
7	318	73	587	68	3224	1114	310	306
7.5	317	58	613	61	3573	940	281	282
8	300	62	580	67	3303	919	271	304
8.5	289	55	562	64	3070	825	260	266
9	286	66	580	65	2713	927	217	244
9.5	273	56	546	56	2516	895	207	219
10	254	42	517	45	2434	872	205	208
10.5	232	31	464	43	2233	840	243	215
11	212	39	446	48	1915	764	187	205
11.5	199	50	445	55	1516	731	154	193
12	196	66	459	67	1382	658	141	187
24	105	66	459	44	1639	1087	105	631

Time (hr)	HMGCS	LSS	CYP51A1	TM7SF2	SC4MOL	NSDHL	HSD17B7	DHCR24
0	1441	77	76	396	4770	43	261	136
0.5	1510	72	74	402	4570	44	253	117
1	1707	85	72	391	4931	52	254	115
1.5	1809	92	69	373	5256	63	258	108
2	1649	98	60	312	5158	68	242	102
2.5	1389	90	61	299	4859	72	250	97
3	1307	88	61	287	3901	79	238	91
3.5	1502	85	67	323	3688	77	255	89
4	1538	87	61	296	3320	59	216	84
4.5	1665	81	62	322	3169	52	208	84
5	1309	79	65	319	2887	58	173	89
5.5	1101	74	67	344	2359	68	169	90
6	853	71	65	314	2056	65	155	89
6.5	877	74	64	330	1875	64	169	84
7	928	74	66	341	1814	67	173	90
7.5	893	72	63	347	1832	63	173	90
8	799	68	55	319	1694	59	163	84
8.5	678	60	51	307	1615	56	160	77
9	628	57	50	293	1603	58	149	74
9.5	672	56	54	276	1689	63	151	79
10	710	59	52	260	1764	65	139	81
10.5	724	56	53	247	1659	66	150	76
11	605	53	53	248	1460	61	140	73
11.5	558	47	55	227	1428	56	139	63
12	574	52	63	225	1426	59	133	69
24	301	44	22	225	1334	21	149	140

Time (hr)	EBP	SC5DL
0	1079	601
0.5	1533	583
1	1505	664
1.5	1380	771
2	1258	809
2.5	1192	792
3	1277	693
3.5	1402	757
4	1402	734
4.5	1359	739
5	1182	623
5.5	1244	525
6	1272	452
6.5	1497	459
7	1628	500
7.5	1709	471
8	1778	465
8.5	1691	413
9	1655	435
9.5	1613	431
10	1647	463
10.5	1659	454
11	1493	417
11.5	1298	372
12	1224	359
24	1224	359

6 Normalized enzyme activity time course following treatment with IFNγ.

Time (hr)	HMGCR	MVK	PMVK	MVD	FDPS	FDFT1	SQLE	DHCR7
0	270	87	925	130	7076	2892	677	635
0.5	296	75	902	134	6391	3508	647	577
1	277	83	886	149	7127	3302	768	680
1.5	320	84	1016	137	7821	3668	810	661
2	288	94	1014	140	7617	2795	811	753
2.5	286	74	932	109	5545	2561	648	568
3	245	64	821	96	4392	1956	539	537
3.5	247	55	872	69	4355	1934	464	423
4	271	66	1107	69	5263	1834	407	409
4.5	254	69	1193	72	5082	1678	375	372
5	258	87	1347	89	5306	1579	344	360
5.5	247	78	1299	87	4728	1419	326	323
6	241	85	1301	90	5479	1175	271	311
6.5	228	66	1190	81	5239	928	331	252
7	211	63	1169	81	5324	807	203	259
7.5	207	55	1110	75	4373	745	197	212
8	219	67	1003	88	3942	756	188	206
8.5	224	80	957	84	3840	694	172	168
9	219	80	834	79	3189	600	152	157
9.5	190	72	857	74	2811	515	159	162
10	174	57	789	64	2264	465	167	166
10.5	186	70	778	71	2227	527	200	178
11	186	69	679	60	1993	521	205	173
11.5	178	67	609	61	1833	496	202	182
12	156	58	602	62	1544	450	185	180
24	99	58	602	84	3870	1667	345	631

Time (hr)	HMGCS	LSS	CYP51A1	TM7SF2	SC4MOL	NSDHL	HSD17B7	DHCR24
0	2594	93	109	419	5539	78	227	152
0.5	2491	104	117	410	5657	89	242	131
1	2527	104	120	414	5593	74	270	138
1.5	1930	99	151	497	6129	70	324	122
2	1740	92	150	494	5924	63	326	123
2.5	1364	85	129	422	5303	68	247	105
3	1663	75	97	334	4205	59	201	89
3.5	1803	65	94	331	3805	51	185	77
4	1915	66	116	397	3919	50	224	84
4.5	1437	65	123	406	3693	50	226	90
5	1131	71	127	435	3439	61	219	97
5.5	966	65	120	421	3130	57	200	86
6	814	71	138	438	2694	66	186	85
6.5	581	64	145	425	2324	60	172	87
7	476	61	153	427	2028	63	159	92
7.5	500	55	146	420	1923	59	138	102
8	542	57	172	448	1849	60	146	99
8.5	545	57	202	475	1676	64	146	96
9	501	58	203	450	1714	66	153	89
9.5	513	56	175	420	1726	67	144	83
10	535	49	145	367	1811	60	130	77
10.5	607	58	109	358	1962	68	136	83
11	643	50	75	332	2218	63	122	67
11.5	662	50	52	313	2402	60	118	64
12	679	41	49	283	2421	57	101	55
24	1221	58	65	283	5596	80	136	140

Time (hr)	EBP	SC5DL
0	1646	870
0.5	1696	908
1	1815	911
1.5	2303	1026
2	2444	995
2.5	2033	899
3	1671	731
3.5	1610	694
4	1870	683
4.5	1944	662
5	1924	638
5.5	1914	657
6	2035	606
6.5	2202	511
7	2239	468
7.5	2233	460
8	2020	512
8.5	1953	499
9	1703	454
9.5	1703	378
10	1780	319
10.5	1820	302
11	1902	290
11.5	1805	274
12	1848	243
24	1848	243

7 Calculating initial conditions

The most general case is an interaction in which the substrate is consumed in a Michaelis-Menten interaction and an off-pathway, competing mass action interaction. If F is the rate at which a substrate is formed, the pathway will be at dynamic equilibrium if

$$F = \frac{k_{cat}ES}{k_m + S} + k_b S$$

where k_b is the mass action rate constant for the off-pathway interaction. After some rearrangement, this yields the following quadratic equation

$$0 = k_b S^2 + (k_b k_m + k_{cat}E - F)S - Fk_m$$

which, from the monotonicity of the mass action term and the Michaelis-Menten terms, can be seen to have one positive solution.

Where an interaction is mass action in form, we have dynamic equilibrium when

$$F = k_{autocat}S + k_b S$$

This can be rearranged to

$$S = \frac{F}{k_{autocat} + k_b}$$

Zymosterol is consumed in two Michaelis-Menten interactions. Here, dynamic equilibrium is established when

$$F = \frac{k^1_{cat}E^1 S}{k^1_m + S} + \frac{k^2_{cat}E^2 S}{k^2_m + S} + k_b S$$

After some rearrangement, this yields the following cubic equation

$$\begin{aligned} 0 \quad = \quad & k_b S^3 \\ & (k_b k^1_m + k_b k^2_m + k^2_{cat}E + k^1_{cat}E - F)S^2 \\ & (k^2_m k^1_m k_b + k^1_c E k^2_m + k^2_c E k^1_m - Fk^1_m - Fk^2_m)S \\ & -Fk^1_m k^2_m \end{aligned}$$

which, from the monotonicity of the two Michaelis-Menten terms and the mass action term, can be seen to have one positive solution.

8 Metabolite levels at 0, 12 and 24 hours post infection or post treatment

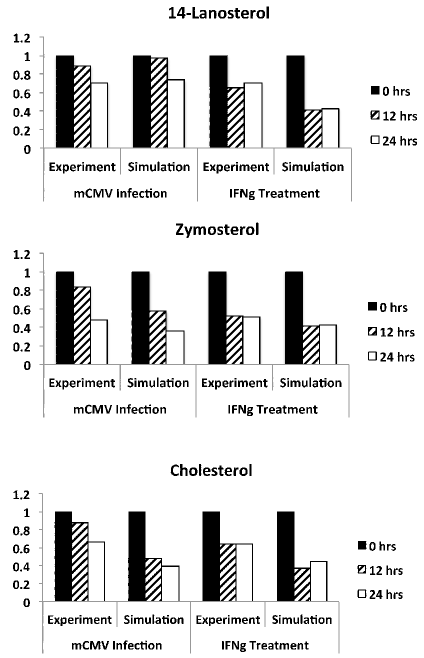

Figure 1: **Metabolite levels at 0, 12 and 24 hours post infection or post treatment.** The normalized concentrations of 14-Lanosterol, Zymosterol and Cholesterol at 0 hours (solid, black), 12 hours (diagonal stripes) and 24 hours (open boxes) after mCMV infection and after IFNγ treatment. We show results from experiment and simulation. Experimental measurements were normalized against measurements from a mock time course and simulated measurements were normalized against the concentration at 0hrs.

9 Inhibitor levels responsible for the flux profile in Fig. 4B

Interaction	Inhibitor to K_i ratio
ACoA-HCoA	22.94
HCoA-M	23.97
M-M5P	23.91
M5P-M5PP	24.00
M5PP-IsPP	23.98
IsPP-FPP	24.00
FPP-Squa	24.00
Squa-23Ox	23.57
23Ox-Lan	23.98
Lan-44Di	23.99
44Di-14De	24.00
14De-4MZC	24.00
4MZC-3K4M	23.99
3K4M-4MZ	23.87

10 Flux profiles from 0 to 24 hours post IFNγ treatment

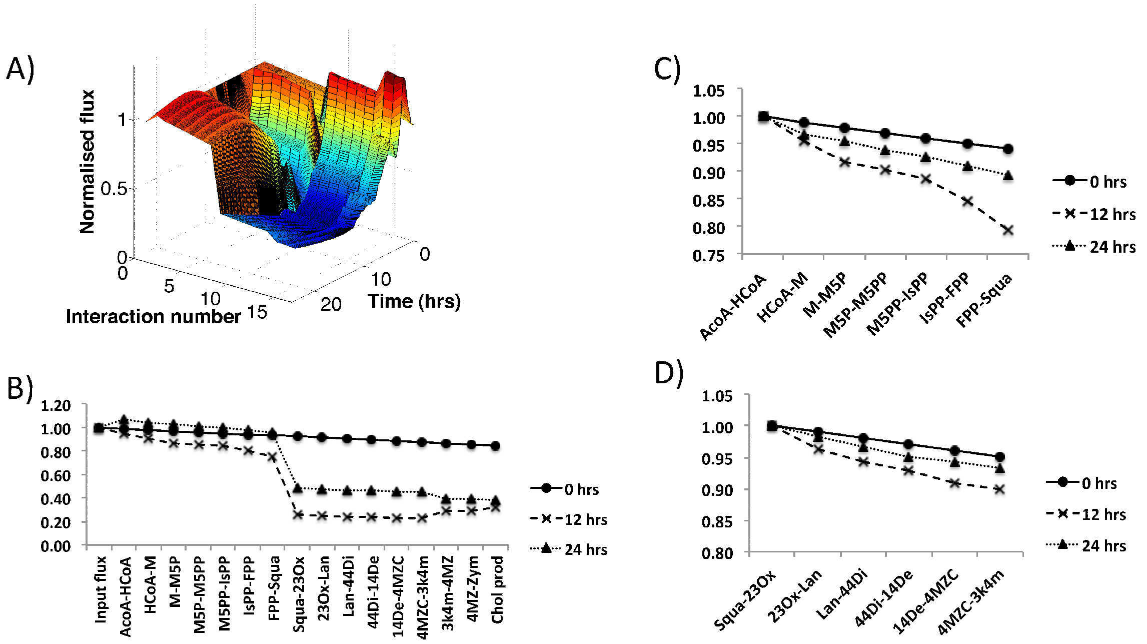

Figure 2: **Flux profiles from 0 to 24 hours post IFNγ treatment.** The flux through the cholesterol biosynthesis pathway following treatment with IFNγ. A) The development of the flux through the pathway in simulation is shown from 0 hrs to 24 hrs following treatment. Interactions are numbered from 1 (the input flux) to 17 (cholesterol production). For the full numbering, see Supplementary section 12. At 0 hrs, the flux through the pathway is relatively constant. However, by 12 hrs the flux has been significantly suppressed along the pathway. By 24 hrs, the pathway has started to show a modest recovery. B) The profile of flux through the pathway at 0 hrs, 12 hrs and 24 hrs following treatment. These profiles represent cross sections of the surface shown in A). The flux is dramatically reduced in the first 12 hrs with a modest increase occurring between 12 and 24 hrs following treatment. Interactions can be classified as dominant (Squa-23Ox) and non-dominant (the remainder) depending on their degree of impact on the pathway flux. C) The flux through the non-dominant interactions between ACoA-HCoA and FPP-Squa, normalized against the flux through the ACoA-HCoA interaction. D) The flux through the non-dominant interactions between Squa-23Ox and 4MZC-3K4M normalized against the flux through the interaction Squa-23Ox.

11 Flux profiles from 0 to 24 hours post mCMV infection

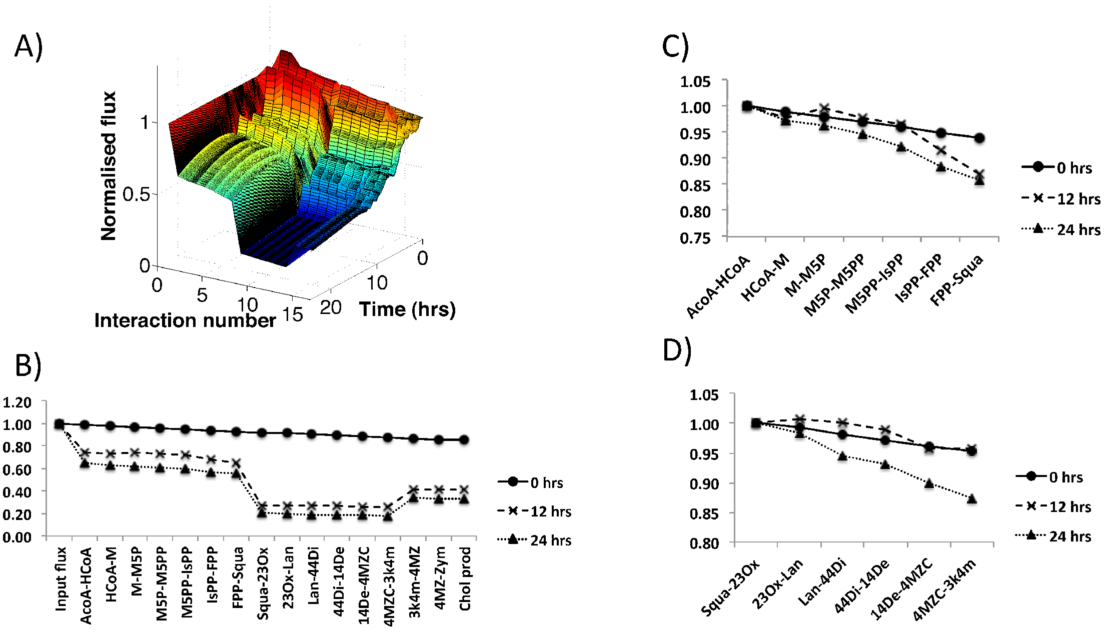

Figure 3: **Flux profiles from 0 to 24 hours post mCMV infection.**The flux through the cholesterol biosynthesis pathway following infection with mCMV. A) The development of the flux through the pathway in simulation is shown from 0 hrs to 24 hrs post infection. Interactions are numbered from 1 (the input flux) to 17 (cholesterol production). For the full numbering, see Supplementary section 12. At 0 hrs, the flux through the pathway is relatively constant. However, by 24 hrs the flux has been significantly suppressed along the pathway. B) The profile of flux through the pathway at 0 hrs, 12 hrs and 24 hrs post infection. These profiles represent cross sections of the surface shown in A). The flux is dramatically reduced in the first 12 hrs with a further reduction occurring between 12 and 24 hrs following infection. Interactions can be classified as dominant (ACoA-HCoA and Squa-23Ox) and non-dominant (the remainder) depending on their degree of impact on the pathway flux. C) The flux through the non-dominant interactions between ACoA-HCoA and FPP-Squa, normalized against the flux through the ACoA-HCoA interaction. The flux through these non-dominant interactions shows a mild suppression between 0 hrs and 12 hrs, but a more significant suppression between 12 hr sand 24 hrs. D) The flux through the non-dominant interactions between Squa-23Ox and 4MZC-3K4M normalized against the flux through the interaction Squa-23Ox. The flux through these interactions shows no suppression between 0 hrs and 12 hrs, but a significant suppression between 12 hrs and 24 hrs.

12 Interaction numbering

1	Input flux
2	AcoA-HCoA
3	HCoA-M
4	M-M5P
5	M5P-M5PP
6	M5PP-IsPP
7	IsPP-FPP
8	FPP-Squa
9	Squa-23Ox
10	23Ox-Lan
11	Lan-44Di
12	44Di-14De
13	14De-4MZC
14	4MZC-3k4m
15	3k4m-4MZ
16	4MZ-Zym
17	Cholesterol production (7DeC-Chol + Des-Chol)
